# Geriatric syndromes and subsequent health-care utilization among older community dwellers in Stockholm

**DOI:** 10.1007/s10433-021-00600-2

**Published:** 2021-01-18

**Authors:** Jette Möller, Christian Rausch, Lucie Laflamme, Yajun Liang

**Affiliations:** 1 Department of Global Public Health, Karolinska Institutet, Widerströmska, 17177 Stockholm, Sweden; 2 Department of Health Sciences, Community and Occupational Medicine, University Medical Center Groningen, University of Groningen, Groningen, The Netherlands

**Keywords:** Geriatric syndromes, Inpatient care, Outpatient care, Polypharmacy

## Abstract

Little is known about the long-term effect of geriatric syndromes on health-care utilization. This study aims to determine the association between geriatric syndromes and health-care utilization during a four-year period among older community dwellers. Based on the Stockholm Public Health Cohort study, a total number of 6700 community dwellers aged ≥65 years were included. From a baseline survey in 2006, geriatric syndromes were defined as having at least one of the following: insomnia, functional decline, urinary incontinence, depressive symptoms and vision impairment. Health-care utilization was identified by linkages at individual level with register data with a four-year follow-up. Cox regression was performed to estimate the associations. Compared to those without geriatric syndromes, participants with any geriatric syndromes had a higher prevalence of frequent hospitalizations, long hospital stays, frequent outpatient visits and polypharmacy in each of the follow-up years. After controlling for covariates, having any geriatric syndromes was associated with higher levels of utilization of inpatient and outpatient care as well as polypharmacy. The association was stable over time, and the fully adjusted hazard ratio (95% confidence interval) remained stable in frequent hospitalizations (from 1.89 [1.31, 2.73] in year 1 to 1.70 [1.23, 2.35] in year 4), long hospital stay (from 1.75 [1.41, 2.16] to 1.49 [1.24, 1.78]), frequent outpatient visits (from 1.40 [1.26, 1.54] to 1.33 [1.22, 1.46]) and polypharmacy (from 1.63 [1.46, 1.83] to 1.53 [1.37, 1.71]). Having any geriatric syndromes is associated with higher levels of health-care utilization among older community dwellers, and the impact of geriatric syndromes is stable over a four-year period.

**Supplementary Information:**

The online version contains supplementary material available at. 10.1007/s10433-021-00600-2.

## Introduction

Geriatric syndromes are increasingly becoming a focus of attention in research due to their high burden on healthy ageing (Prince et al. 2015). Geriatric syndromes are considered to be indicators of ageing-related dysfunctions spanning across different organ systems (Inouye et al. 2007). The presence of geriatric syndromes tends to indicate pathological ageing, higher susceptibility to stressors like diseases and decreased capacity for rehabilitation (Wang et al. 2013). So far, there is no consensus on the definition of geriatric syndromes; however, most previous studies have defined geriatric syndromes as the presence of at least one of the following conditions: pressure ulcers, incontinence, functional decline, delirium, insomnia, hearing or vision problem, depressive symptoms and falls (Inouye et al. 2007; Skalska et al. 2013; Liang et al. 2018). These commonly presented items during the ageing process are associated with negative health outcomes, e.g. multimorbidity, frailty, physical disability, brain white matter disease and low life satisfaction (Marengoni et al. 2011; Prince et al. 2015; Rizzuto et al. 2017; Rosso et al. 2013; Yang et al. 2015; Alagiakrishnan et al. 2013; Lane et al. 2017).

Furthermore, as geriatric syndromes indicate higher susceptibility to stressors and inability to rehabilitate, older people may require more health care to maintain an adequate level of functioning. In spite of the high prevalence of geriatric syndromes and in view of their likely co-occurrence with other health conditions, surprisingly little is known about their link to health-care utilization, measured in terms of, for example, outpatient clinic attendance, hospitalizations, emergency visits or medication use, except for recent studies from Asia and a meta-analysis (Cheung et al. 2018; Chiu and Cheng 2019; Vermeiren et al. 2016). In Hong Kong, a study among community-dwelling older adults showed that geriatric syndromes alone yield more health-care utilization (e.g. more visits to general outpatient and specialist outpatient clinics) than multimorbidity alone (Cheung et al. 2018). In Taiwan, it was observed that some (e.g. incontinence, falls and pain) but not all geriatric syndromes correlated equally strongly with health-care utilization (Chiu and Cheng 2019). Regarding the fact that geriatric syndromes may both coexist and share underlying mechanisms, it may be more indicative to study them as a group rather than individually (Cheung et al. 2018). Studies on the association between geriatric syndromes and health-care utilization are limited, and no study has considered the temporal association between the presence of geriatric syndromes and the progression of health-care utilization over time (Cheung et al. 2018; Chiu and Cheng 2019). Studies of this association are motivated in the light of the growing number of older people, the high prevalence of geriatric syndromes among them (Liang et al. 2018) and the likely impoverishment of health over time once these syndromes have occurred (Lane et al. 2019).

In this study, we aim to examine the association between geriatric syndromes and health-care utilization among older community dwellers and to determine whether it changes over time.

## Methods

### Study design and participants

This study was based on data from the sub-cohort of the Stockholm Public Health Cohort (SPHC). The SPHC is an ongoing cohort study, which was initiated and conducted for the purpose of health surveillance and risk factor assessment as well as for the formulation, planning and evaluation of health policy (Svensson et al. 2013). Three cohorts in SPHC have been identified including cohorts 2002, 2006 and 2010. For each cohort, at baseline a study sample was selected based on an area-stratified randomization, sampling from the eligible population of Stockholm County including adults aged 18–84 years in 2006 and aged ≥18 years in 2010. For the purpose of this study, we included the sub-cohort 2006, which was investigated in 2006 at baseline and followed for 4 years for the register information on health-care utilization. In 2006, a total of 56,634 participants received the questionnaire and 34,707 responded, corresponding to a response rate of 61.3% (Svensson et al. 2013). We excluded those aged <65 years of age (*n* = 27,994) and those with missing information on geriatric syndromes (*n* = 13), resulting in a total study population of 6700 participants (19.3%) aged 65 years and above with information on geriatric syndromes at baseline.

### Data collection and definitions

Data were collected through post-based questionnaires as well as linked to information from Swedish health registers, i.e. Longitudinal Integrated Database for Health Insurance and Labour Market Studies (LISA, with socio-demographic information since 1990) (Statistics Sweden), the National Patient Register (NPR, covering the national inpatient care since 1987 and outpatient specialist care since 2001) and the Swedish Prescribed Drug Register (SPDR, including all dispensations of prescribed medications since 2005) (The National Board of Health and Welfare).

### Exposures

Geriatric syndromes were defined as suffering from any of the following five conditions: insomnia, functional dependence, urinary incontinence, depressive symptoms and vision impairment as per self-reported data in the questionnaires. Insomnia was defined as having light to heavy sleeping problems. Functional dependence was defined as being unable to walk or run 100 m or use stairs. Urinary incontinence was defined as having light to heavy urine leakage. Based on a 12-item general health questionnaire, depressive symptoms were defined as having a score of 3 and above (Kim et al. 2013). Vision impairment was defined as having difficulty in reading or distinguishing text in a newspaper even with glasses.

### Outcomes

Health-care utilization was assessed according to number of hospital visits, hospitalized days, outpatient visits and medications annually during follow-up (Axmon et al. 2016). From the Swedish NPR, we identified and calculated the number of hospitalizations and the number of days in hospital for each year. Frequent hospitalizations were defined as an annual number of three or more hospitalizations. Long hospital stay was defined as three or more annual number of days in hospital. Frequent outpatient visits were defined as having more than 10 visits in outpatient specialized care on an annual basis. Based on the SPDR, number of medications was calculated based on the Anatomical Therapeutic Chemical Classification System (ATC) code of five letters during the period of a year. The SPDR covers all dispensations of prescribed medications at pharmacies in Sweden, however, no medications administered during hospitalization or over-the-counter medications. Polypharmacy was defined as having 5 or more medications dispensed within the same year.

### Covariates

Data on demographic factors (e.g. age, sex, marital status and country of birth) were collected through post-based questionnaires. Marital status was categorized into married, unmarried, divorced and widowed. Country of birth was grouped into Swedish-born and non-Swedish-born. Multimorbidity at baseline was defined as having at least two diagnoses of any diseases based on diagnoses in the patient register one year prior to baseline.

### Statistical analysis

Chi-square tests were performed to compare the baseline characteristics between participants with any geriatric syndromes and those without. Prevalence (%) and 95% confidence interval (CI) of health-care utilization (e.g. frequent hospitalizations, long hospital stay, frequent outpatient visits, polypharmacy) were presented for each year for all participants and in subgroups by geriatric syndromes (Online Resource 1). The prevalence was graphed by years of follow-up for those with and without geriatric syndromes, respectively. Cox regression was performed to assess the association between geriatric syndromes and health-care utilization for each year. The time variable in Cox regression models was included as 1, 2, 3 and 4 for the first, second, third and fourth year, respectively. Three models were performed: model 1 was a crude model; model 2 was adjusted for age, sex, marital status and country of birth; and model 3 was further adjusted for multimorbidity. Hazard ratio (HR) and 95% CI from the three models were presented for the associations between geriatric syndromes and health-care utilization.

IBM SPSS Statistics 26 for Windows (IBM SPSS Inc., Chicago, Illinois, USA) was used for all analyses.

## Results

At baseline, there were 5118 participants (76.4%) defined to have at least one geriatric syndrome. The prevalence of specific geriatric syndrome items was 59.9% for insomnia, 37.2% for functional dependence, 28.1% for urinary incontinence, 11.1% for depressive symptom and 4.7% for vision impairment. The prevalence for having no geriatric syndromes, one geriatric syndrome item and ≥2 items was 23.6%, 35.4% and 41.0%, respectively.

Table 1 shows the comparison of baseline characteristics between participants with and those without any geriatric syndromes. Compared with participants without geriatric syndromes, those with geriatric syndromes were more often women (*p* < 0.001), divorced or widowed (*p* < 0.001), non-Swedish-born (*p* = 0.001) and more likely to have multimorbidity (*p* < 0.001).

**Table 1:** Baseline characteristics of the study population stratified by geriatric syndromes at baseline

Characteristics	Total *n* = 6700	No geriatric syndromes *n* = 1582	Any geriatric syndromes *n* = 5118	*p*
*Sex, %*				<0.001
Men	45.3	60.3	40.7	
Women	54.7	39.7	59.3	
*Marital status, %*				<0.001
Married	56.3	62.0	54.5	
Unmarried	6.7	7.8	6.4	
Divorced	17.7	16.7	18.0	
Widowed	19.3	13.5	21.1	
*Swedish-born, %*				0.001
Yes	82.4	85.2	81.5	
No	17.6	14.8	18.5	
*Multimorbidity, %*	6.0	1.9	7.2	<0.001

**Fig. 1:**
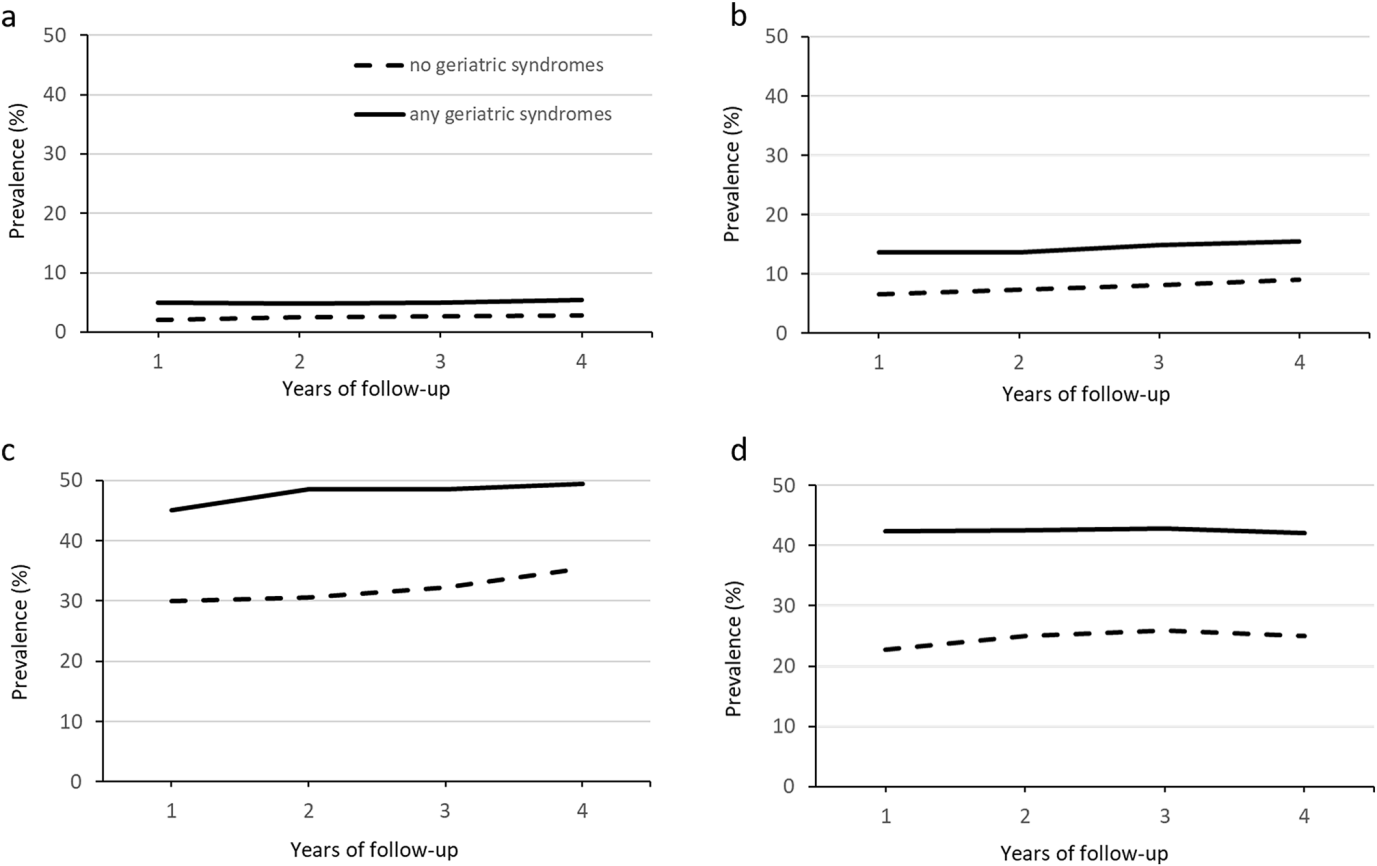
Prevalence of health-care utilization by year of follow-up stratified by geriatric syndromes at baseline and by type of utilization. **a** frequent hospitalizations; **b** long hospital stay; **c** frequent outpatient visits; **d** polypharmacy

In Fig. 1 and Online Resource 1, the prevalence (95% CI) of health-care utilization is described for those with and without geriatric syndromes across the follow-up years. Compared to those without geriatric syndromes, participants with geriatric syndromes had higher prevalence of frequent hospitalizations, long hospital stay, frequent outpatient visits and polypharmacy in each year of the follow-ups. In the total sample, the prevalence (95% CI) remained stable in frequent hospitalizations (from 4.3 [3.9, 4.8] in year 1 to 4.8 [4.3, 5.3] in year 4) and polypharmacy (from 37.8 [36.6, 39.0] in year 1 to 38.1 [36.9, 39.2] in year 4), but increased over time in long hospital stay (from 11.9 [11.1, 12.7] to 14.0 [13.2, 14.8]) and frequent outpatient visits (from 41.5 [40.4, 42.7] to 46.2 [45.0, 47.4]). The long hospital stay increased only among those with geriatric syndromes, whereas the increase in frequent outpatient visits was found in both groups.

The most common main diagnoses for the hospitalizations were circulatory system diseases (21.3%), and most common diagnoses of outpatient visits were health service and medical care due to different reasons (17.8%), e.g. lifestyle counselling, follow-up examinations, cancer treatment and functional implants.

Table 2 shows the association between geriatric syndromes and health-care utilization over time. During each year, geriatric syndromes were associated with a higher level of all health-care utilization: frequent hospitalizations, long hospital stays, frequent outpatient visits and polypharmacy in all three models. Over time, the association between geriatric syndromes and health-care utilization did not change significantly, and the fully adjusted HR (95% CI) remained stable across 4 years of follow-up.

**Table 2:** Association between geriatric syndromes and annual health-care utilization over time

	Hazard ratio (95% confidence interval)^a^			
Follow-up time	Frequent hospitalizations	Long hospital stay	Frequent outpatient visits	Polypharmacy
*First year*				
Model 1	2.34 (1.63, 3.34)	2.07 (1.68, 2.54)	1.51 (1.36, 1.66)	1.86 (1.66, 2.08)
Model 2	2.15 (1.50, 3.10)	1.88 (1.52, 2.31)	1.43 (1.29, 1.58)	1.68 (1.50, 1.88)
Model 3	1.89 (1.31, 2.73)	1.75 (1.41, 2.16)	1.40 (1.26, 1.54)	1.63 (1.46, 1.83)
*Second year*				
Model 1	1.80 (1.30, 2.50)	1.87 (1.54, 2.27)	1.58 (1.44, 1.74)	1.70 (1.53, 1.90)
Model 2	1.61 (1.15,2.25)	1.67 (1.37, 2.04)	1.48 (1.34, 1.64)	1.54 (1.38, 1.71)
Model 3	1.49 (1.06, 2.09)	1.59 (1.30, 1.95)	1.46 (1.33, 1.62)	1.52 (1.36, 1.69)
*Third year*				
Model 1	1.83 (1.32, 2.52)	1.81 (1.51, 2.18)	1.51 (1.37, 1.66)	1.66 (1.49, 1.84)
Model 2	1.59 (1.15, 2.22)	1.60 (1.32, 1.93)	1.41 (1.28, 1.55)	1.51 (1.36, 1.68)
Model 3	1.50 (1.08, 2.09)	1.55 (1.28, 1.87)	1.40 (1.27, 1.54)	1.49 (1.34, 1.66)
*Fourth year*				
Model 1	1.95 (1.42, 2.68)	1.72 (1.44, 2.05)	1.40 (1.28, 1.53)	1.69 (1.52, 1.88)
Model 2	1.76 (1.28, 2.44)	1.52 (1.27, 1.82)	1.33 (1.22, 1.46)	1.54 (1.38, 1.72)
Model 3	1.70 (1.23, 2.35)	1.49 (1.24, 1.78)	1.33 (1.22, 1.46)	1.53 (1.37, 1.71)

^a^Model 1 was a crude model, model 2 was adjusted for age, sex, marital status and birth country, and model 3 was additionally adjusted for multimorbidity at baseline

## Discussion

In this study, we found that having geriatric syndromes is linked to higher levels of health-care utilization such as frequent hospitalizations, long hospital stays, frequent outpatient visits and polypharmacy among community-dwelling older adults. The association between geriatric syndromes and health-care utilization remained stable over time suggesting that geriatric syndromes have a long-term and stable impact on health-care need.

Our finding was in line with the findings from the previous studies showing that geriatric syndromes (e.g. incontinence) were associated with a higher risk of subsequent health-care utilization (Chiu and Cheng 2019). Furthermore, we found that the association between geriatric syndromes and health-care utilization was still significant after adjusting for multimorbidity. This result was also consistent with previous findings that the increased attendance rate at outpatient clinics caused by geriatric syndromes was independent of multimorbidity (Cheung et al. 2018). The presence of at least one geriatric syndrome causes a big health burden and hence increases the use of health care, which might be due to the preclinical and clinical pathophysiologic changes (e.g. multisystem dysregulation, inflammation, sarcopenia and atherosclerosis) underlying several co-occurring conditions (Inouye et al. 2007; Skalska et al. 2013; Alagiakrishnan et al. 2013) that require more frequent attention or longer stays in hospital due to decreased recovery abilities.

In older adults without geriatric syndromes, we found that the prevalence of frequent outpatient care increased over time, which might be explained by the consequences of the ageing process (Christensen et al. 2009). During the ageing process, arterial ageing plays an important role in primary pathological mechanisms of ageing-related endothelial dysfunction (e.g. oxidative stress and inflammation), decrease in hormone activity and the decline in bodily functions (Tesauro et al. 2017; van den Beld et al. 2018). In those with any geriatric syndromes, the increased utilization of inpatient and outpatient care might be explained by the ageing process in combination with geriatric syndromes. However, it is difficult to disentangle the ageing-induced effects from the impact of geriatric syndromes and other factors (e.g. inflammation and chronic diseases), all of which may also affect the utilization of health care (van den Beld et al. 2018).

In addition, we found that the prevalence of polypharmacy remained stable over time with a prevalence of 38%–39%. This was comparable with that from the previous study (39.1%) among community-dwelling persons aged ≥65 years in Sweden (Johnell and Fastbom 2012). However, we did not observe any change in prevalence of polypharmacy over time in total participants as well as in the two groups by geriatric syndromes. It is possible that the stable prevalence of polypharmacy is due to the guidelines for regular medication reviews (Lenander et al. 2014). Since we identify medications based on the five-character levels of the ATC codes, the total number of medications in this study might include several medicines within one type. During the medication review, the total number of medications is often assessed, and if a new one is added, an old one especially of the same type is taken out, which might result in a stable prevalence of polypharmacy.

Furthermore, we found that the effect of geriatric syndromes remained stable over time, which implies that geriatric syndromes have a stable impact on health-care utilization over a four-year period. Our findings have important public health implications, namely that geriatric syndromes cannot simply be neglected as a part of becoming old, and their early prevention, detection and intervention are necessary for reducing the need of future health care. However, due to the lack of comparable measurements between baseline and follow-up, we were not able to take into account changes in geriatric syndromes during the follow-up period. Since some of the geriatric syndromes’ conditions are reversable, the two groups of participants might be exchangeable. This could lead to an overestimation of health-care use in those without geriatric syndromes, but an underestimation of health-care utilization for persons with any geriatric syndromes. Future studies are needed with the variation of geriatric syndromes and the time-varying effect of other contributing factors (e.g. socio-economic conditions) of health-care utilization regarding that there are socio-economic differences in health-care use among Swedish old adults (Wastesson et al. 2014).

This study has several strengths, such as the population-based longitudinal study design, the large sample size and being able to adjust for the potential confounders. Moreover, data on health-care utilization were based on health registers, which included information of all participants without any missing data on health-care utilization over time. However, the study also has limitations that need to be acknowledged. First, geriatric syndromes were assessed based on self-reported information, which might underestimate the prevalence of some specific geriatric syndromes due to stigmatization or recall bias. On the other hand, some geriatric syndromes (e.g. insomnia and less severe depressive symptoms) cannot be captured without asking directly since information is not available in health registers. Second, the non-respondents (~40%) to the postal questionnaires at baseline are more likely to be male, younger, born outside of Sweden and to have a lower education and income (Svensson et al. 2013). People with mental health problems are less likely to participate than those without, which might lead to a lower prevalence of depressive symptom in our study. In addition, regarding that the prevalence of geriatric syndromes was lower in younger elderly and in males but higher in those with lower education and born outside Sweden (Liang et al. 2018), thus, the non-respondents might affect the prevalence of geriatric syndromes in both directions. The generalizability of our findings to the whole population should be approached with caution. Furthermore, death and migration were not taken into consideration to describe the prevalence of health-care utilization, which might underestimate the effect of geriatric syndromes on the use of health care.

## Conclusions

Having any geriatric syndromes is associated with higher levels of health-care utilization, and the impact of geriatric syndromes on health care remains stable over time. The study implies that geriatric syndromes have a long-term impact on health care, and therefore, the findings should be considered in the development of effective care delivery strategies through early identification and management of geriatric syndromes.

## Supplementary Information

Below is the link to the electronic supplementary material.

Supplementary file1 (DOCX 22 kb)

## Data Availability

Data are from the Stockholm Public Health Cohort (SPHC), study and access to the data is available upon approval by the SPHC data management committee. (https://www.folkhalsoguiden.se/halsa-stockholm/halsa-stockholm-for-forskare).
