# Supplementary material for: Geriatric syndromes and subsequent health-care utilization among older community dwellers in Stockholm
Source: Eur J Ageing. 2021 Jan 18;19(1):19–25. doi: 10.1007/s10433-021-00600-2 (PMC8881534; doi:10.1007/s10433-021-00600-2)
Supplement: Supplementary file 1 — Supplementary file1 (DOCX 22 kb) [file 10433_2021_600_MOESM1_ESM.docx]

**Online Resource 1** Prevalence of health-care utilization by year of follow up stratified by geriatric syndromes at baseline and by type of utilization

| Follow-up year | Prevalence (95% confidence interval) | | | |
| --- | --- | --- | --- | --- |
|  | Frequent hospitalizations | Long hospital stay | Frequent outpatient visits | Polypharmacy |
| Frist year |  |  |  |  |
| Total | 4.3 (3.9, 4.8) | 11.9 (11.1, 12.7) | 41.5 (40.4, 42.7) | 37.8 (36.6, 39.0) |
| No geriatric syndromes | 2.1 (1.4, 2.9) | 6.6 (5.4, 7.8) | 30.0 (27.7, 32.2) | 22.8 (20.8, 24.9) |
| Any geriatric syndromes | 5.0 (4.4, 5.6) | 13.6 (12.6, 14.5) | 45.1 (43.8, 46.5) | 42.4 (41.1, 43.8) |
| Second year |  |  |  |  |
| Total | 4.3 (3.8, 4.8) | 12.2 (11.4, 13.0) | 44.3 (43.1, 45.5) | 38.5 (37.3, 39.6) |
| No geriatric syndromes | 2.6 (1.9, 3.4) | 7.3 (6.0, 8.6) | 30.6 (28.4, 32.9) | 25.0 (22.9, 27.2) |
| Any geriatric syndromes | 4.8 (4.2, 5.4) | 13.7 (12.8, 14.6) | 48.5 (47.1, 49.8) | 42.6 (41.3, 44.0) |
| Third year |  |  |  |  |
| Total | 4.4 (3.9, 4.9) | 13.3 (12.5, 14.1) | 44.6 (43.5, 45.8) | 38.9 (37.7, 40.1) |
| No geriatric syndromes | 2.7 (1.9, 3.5) | 8.2 (6.9, 9.6) | 32.2 (29.9, 34.5) | 25.9 (23.8, 28.1) |
| Any geriatric syndromes | 5.0 (4.4, 5.6) | 14.9 (13.9, 15.9) | 48.5 (47.2, 49.9) | 42.9 (41.6, 44.3) |
| Fourth year |  |  |  |  |
| Total | 4.8 (4.3, 5.3) | 14.0 (13.2, 14.8) | 46.2 (45.0, 47.4) | 38.1 (36.9, 39.2) |
| No geriatric syndromes | 2.8 (2.0, 3.6) | 9.0 (7.6, 10.4) | 35.4 (33.0, 37.8) | 25.0 (22.8, 27.1) |
| Any geriatric syndromes | 5.4 (4.8, 6.0) | 15.5 (14.5, 16.5) | 49.5 (48.2, 50.9) | 42.1 (40.8, 43.5) |
